# Geometric consistency among atherosclerotic plaques in carotid arteries evaluated by multidimensional parameters

**DOI:** 10.1016/j.heliyon.2024.e37419

**Published:** 2024-09-06

**Authors:** Ling Li, Fangyu Dai, Jie Xu, Jiaoxuan Dong, Bin Wu, Songbin He, Haipeng Liu

**Affiliations:** a Department of Neurology, Zhoushan Hospital, Wenzhou Medical University, Zhoushan, 316000, Zhejiang Province, China; b Department of Medicine and Therapeutics, The Chinese University of Hong Kong, Hong Kong, Hong Kong Special Administrative Region of China; c Department of Neurology, People's Hospital of Quzhou, Quzhou, 324002, Zhejiang Province, China; d Department of Neurology, Zhoushan Hospital, Zhejiang University, School of Medicine, Zhoushan, 316000, Zhejiang Province, China; e Centre for Intelligent Healthcare, Coventry University, Coventry, CV1 5RW, UK

**Keywords:** Stroke, Carotid atherosclerotic plaque, Computed tomography angiography (CTA), Three-dimensional reconstruction, Plaque morphology

## Abstract

The three-dimensional (3D) geometry of carotid atherosclerotic plaques is associated with multiple cardiovascular diseases. However, it is unknown if carotid plaques of different sizes are consistent in 3D geometry, with a lack of quantitative observation. We aim to evaluate the geometric consistency of carotid plaques using the correlations between multidimensional parameters.

42 cases with asymptomatic stenosis caused by atherosclerotic plaque in the carotid artery were included. Carotid plaques and calcifications were identified on computed tomography angiography images and 3D reconstructed. Multidimensional geometric parameters (length, surface area, volume, etc.) were measured on the reconstructed 3D structures. Linear and non-linear (power function) fittings were used to investigate the relationships between multidimensional parameters. The analysis was performed based on cases and plaques, respectively. Spearman rank correlation analysis, R-squared, and p-values were used to evaluate the significance of the relationship. Significant relationship was defined as R-squared >0.25 and p < 0.05.

In total, 112 atherosclerotic plaques and 74 calcifications were extracted. In plaque-based analysis, significant correlations were widely observed between paired multidimensional parameters of carotid plaques, where non-linear fitting showed higher R-squared values. Plaque volume and surface area were significantly correlated with total volume and total surface area of intra-plaque calcifications. In subject-based analysis, triglycerides and total cholesterol were significantly correlated with carotid plaque size.

There is a consistency in geometry among carotid atherosclerotic plaques of different sizes. The size of a carotid plaque is associated with the patient's lipid profile.

## 1 Introduction

Cardiovascular diseases (CVDs) are the leading cause of death globally, representing one fourth of global mortality. Especially, stroke remains the second leading cause of death in the world, with an increasing incidence in many developing countries [1]. Stroke can be hemorrhagic (i.e., the blood supply is weakened by arterial vessel burst) or ischemic (i.e., the artery is blocked) where the latter accounts for 85 % of all stroke cases [2]. A related conditions is transient ischemic attack (TIA), also known as ‘mini-stroke’, where the blood supply to a certain part of the brain is briefly interrupted due to the blockage of small arteries. The pathophysiological process of stroke is chronic with a long pre-symptomatic stage [3]. Thus, primary prevention of stroke focusing on risk factor modification plays a key role in reducing the burden of stroke [4].

The existence of carotid atherosclerotic plaque is a risk factor of ischemic stroke and TIA [5,6], as well as other neurological diseases including dizziness [7,8]. The geometry and morphology of carotid atherosclerotic plaque reflect the progress of whole-body atherosclerosis and overall risk of CVDs [9,10]. The rupture of a carotid atherosclerotic plaque can cause thrombus and embolization into distal intracranial arteries, leading to ischemic stroke and TIA [5]. The risk of plaque rupture depends on the local hemodynamic and biomechanical environments, which are associated to the plaque geometry and morphology [11]. The morphology of different plaque components, e.g., calcification and fibrous tissues, is associated with the risk of plaque rupture [12]. A large-scale (n = 1939) clinical observation found that the presence of irregular carotid plaque independently predicted ischemic stroke [13]. In addition, the shape of calcification in carotid plaques may be associated with the risk of TIA [14]. Some computational simulation studies also suggest that the plaque geometry can influence the risk of rupture on both idealized [15] and patient-specific models [16]. Therefore, carotid plaque geometry can provide valuable information for the early detection and prevention of stroke and other CVDs.

A well-known aspect of plaque formation is that low wall shear stress (WSS) can accelerate the trans-endothelial transport of low-density lipoprotein (LDL), which is the first stage of atherosclerosis [[17], [18], [19]]. Meanwhile, a plaque can change focal hemodynamic environment (e.g., inducing distal low-WSS areas) [18,20] and vascular distribution of inflammatory markers [21,22]. This geometric-hemodynamic-biomechanical interaction shapes the long-term evolution of plaque geometry and may lead to a consistency in some geometric features [11]. It was found that some multi-dimensional geometric parameters of coronary atherosclerotic plaques with different sizes follow power function relationships, which indicates the consistency in geometry among coronary plaques of difference sizes [23]. However, there is lack of a comprehensive, quantitative evaluation of the geometric consistency of carotid plaques.

Many imaging modalities can assess carotid plaque geometry, including computed tomography angiography (CTA), magnetic resonance angiography (MRA), ultrasonography, and positron emission tomography (PET) [24]. MRA has the best accuracy in detecting different plaque components, therefore is the current gold standard for carotid plaque imaging. PET is highly accurate in identifying plaque inflammation but has poor spatial resolution. The ultrasonic imaging has lower resolution compared with MRA and CTA, and is operator-dependent. CTA has high spatial and contrast resolutions and can differentiate plaque components with high accuracy in detecting calcification [24]. With its wide availability, CTA is the most common tool for assessing carotid plaques in clinical practice.

In this study, we aim to investigate if there is a consistency in geometry across carotid plaques with different sizes. The 3-dimensional (3D) plaque geometry was reconstructed from the CTA data for the measurement and analysis of multidimensional parameters. This study paves the way for disclosing the geometric-hemodynamic-biomechanical interaction in the development of carotid atherosclerosis.

## 2 Methods

### 2.1 General information

This study was approved by the Ethics Committee of Zhoushan Hospital. Informed consent was waived due to the retrospective nature of this study. Carotid CTA images of 42 patients with asymptomatic carotid artery stenosis admitted to the Department of Neurology of Zhoushan Hospital from January 2021 to March 2022 were collected. The inclusion criteria were as follows: mild to moderate stenosis (<70 % in diameter) in carotid artery confirmed by carotid ultrasound and CTA; age between 30 and 80 years; no statin use prior to admission. Exclusion criteria include: (1) severe cerebrovascular diseases and related neurological diseases: transient ischemic attack or stroke, subarachnoid hemorrhage, intracranial infection, intracranial tumor, trauma, neurodegenerative disease, carotid dissection, etc.; (2) with heart, liver or renal insufficiency; (3) complicated with chronic diseases, e.g., coagulation dysfunction, lung disease, blood system disease, malignant tumor, autoimmune disease, severe metabolic disease, mental disorder, etc.; (4) the clinical data were incomplete.

### 2.2 Medical history and blood test

Patient-specific clinical data were collected: basic information, conditions of hypertension and diabetes. Venous blood samples were collected on the second day after admission. A full set of blood lipids and uric acid (UA) were detected by LX-20 (Beckman Coulter, Inc., USA) biochemical instrument. The coagulation function was detected by Sysmex CN-6000 automated coagulation analyser (Sysmex Corporation, Kobe, Japan). Vitamin B12 (VB12) and folic acid (FOL) were detected by DXI-800 automatic microparticle chemiluminescence immunoanalyzer (Beckman Coulter, Inc., USA). The reagents used were all provided by the equipment manufacturers. All steps were carried out strictly according to the operating instructions, and the testing process was strictly controlled. The blood test results included total cholesterol (TC), triglyceride (TG), high-density lipoprotein cholesterol (HDL-C), LDL cholesterol (LDL-C), fibrinogen (FIB), VB12, FOL, and UA.

Carotid artery CTA was performed with GE Revolution 256-row spiral CT angiography system (GE, USA). The observer was blinded for the case diagnosis. The quantity, location, and type of atherosclerotic plaques have been confirmed by a radiologist and a neurologist.

### 2.3 Extraction and 3D reconstruction of carotid artery and plaques

**Fig. 1:**
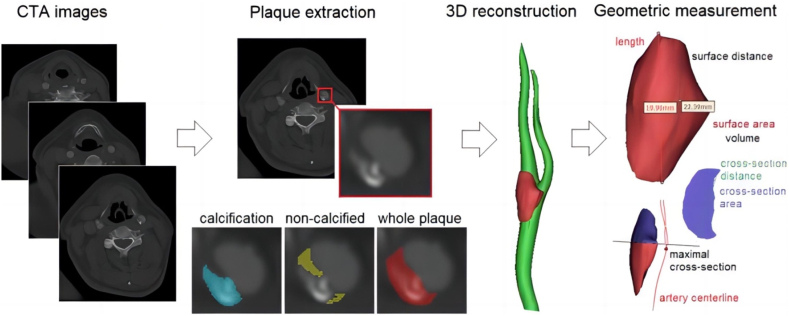
Reconstruction of three-dimensional geometry of atherosclerotic plaques in carotid arteries and the measurement of multidimensional geometric parameters.

The 3D geometry of carotid artery and plaques was reconstructed from the carotid CTA images using the software MIMICS 20.0 (Materialise N.V., Belgium), as shown in Fig. 1. First, bilateral carotid arteries were identified on the transverse (i.e., horizontal) images. The left carotid artery was extracted from the left common carotid artery at the aortic arch to the extracranial segment of the internal carotid artery. The right carotid artery segment was extracted from the right common carotid artery at the innominate artery to the extracranial segment of the internal carotid artery. Then, by setting the boundaries of region of interest in 3D space and the threshold of CT attenuation value (in Hounsfield units, or HU), the carotid artery segments can be automatically extracted and 3D reconstructed, with the result saved in a separate dataset (a "mask" in MIMICS software).

The 3D reconstruction of carotid plaque was based on a semiautomatic two-step approach. Firstly, a plaque was located by specifying its range in the 3D space (i.e., on sagittal, coronal, and transverse planes). Within the specified 3D space, the plaque was automatically extracted using thresholds of CT attenuation value. High (>150-1334 HU) and low (0–150 HU) attenuation values were used to initially identify the calcified and non-calcified components with results saved in two datasets (i.e., “masks”) respectively. The thresholds were adjustable in different plaques considering the differences in scan conditions. Finally, by using union Boolean operation, a mask was developed to cover the whole plaque. Considering the calcification-induced blooming artefact, the masks were visually checked on 2D planes and manually revised by a radiologist to improve the accuracy of the boundaries between calcifications, non-calcified components and artery lumen.

### 2.4 Measurement of multidimensional geometric parameters

The measurement of multidimensional geometric parameters was performed using the Materialise 3Matic software (Materialise N.V., Belgium). For a plaque, the volume and surface area were measured automatically. The length of a plaque was defined as the longest straight-line distance between two points at its proximal and distal ends. The surface distance was defined as the length of the shortest path on the plaque surface between the proximal and distal ends. The cross-sections of the plaque were derived perpendicularly to local artery centreline. The cross-sectional distance (i.e., diameter) and area were measured on the largest cross section. To investigate the longitudinal symmetry of the plaque, we measured the ratio between upstream and downstream lengths, i.e., the ratio between the distances from both (proximal and distal) ends to the largest cross section. Considering the irregular geometry of the calcifications, only the volume and surface area were measured for each calcification. The plaques and calcifications with volume lower than 1 mm^3^ were excluded.

### 2.5 Data analysis

The statistical analysis was performed on SPSS software (Version 26.0, IBM Corp., Armonk, NY, USA) and R programming language (Version 4.3.2, R Core Team 2023). The data analysis consisted of two parts: plaque-based analysis and subject-based analysis.

In the plaque-based analysis, linear and non-linear regressions were performed between multidimensional geometric parameters of plaques: volume, surface area, length, surface distance, cross-sectional area and cross-sectional diameter. The total calcification volume and surface area were calculated for each plaque. To investigate the relationship between the size of a plaque and the progression of calcification, linear regression was carried out between plaque volume and intra-plaque calcification volume (i.e., the total volume of all the calcifications in the plaque), between plaque surface area and intra-plaque calcification surface area. The analysis was performed on all plaques and calcified plaques, respectively. To initially investigate the relationship between plaques’ geometric irregularity and the progression of calcification, linear regression was perform between the total calcification volume and the ratio between plaque volume and plaque surface area. In linear regression, the significant correlation was defined as r > 0.5 (R-squared >0.25) and p < 0.05. The strong correlation was defined as r > 0.8 (R-squared >0.64) and *P* < 0.05. The relationship was further studied by non-linear regression analysis using power function which reflects the relationship between geometric parameters in different dimensions [23].

The subject-based analysis was performed to investigate the relationship between plaque geometry and biochemical factors, especially the levels of lipid materials. Spearman rank correlation analysis was performed between the hematological features and following geometric parameters: total plaque volume (TPV), maximal plaque volume (MPV), total plaque surface area (TPSA), maximal plaque surface area (MPSA), total calcification volume (TCV), and total calcification surface area (TCSA) as well as some secondary parameters: ratio in total volume between calcifications and plaques (RTVCP), ratio in total surface area between calcifications and plaques (RTSACP), the maximum, mean, and minimum values of the ratio between plaque volume and plaque surface area among all plaques (PVSARmax, PVSARmean, PVSARmin), and its value in the largest (i.e., with maximal volume) plaque (PVSARm). Table 1 lists the definitions of the subject-based plaque-related, calcification-related, and secondary parameters.

**Table 1:** Definitions of subject-based plaque-related, calcification-related, and secondary parameters.

Subject-based parameters	Definition
total plaque volume (TPV)	total volume of all carotid plaques in each subject
maximal plaque volume (MPV)	the volume of the largest carotid plaque in each subject
total plaque surface area (TPSA)	summation of all carotid plaque surface areas in each subject
maximal plaque surface area (MPSA)	the maximal value of surface area among all plaques in a subject
total calcification volume (TCV)	total volume of all calcifications in carotid plaques in each subject
total calcification surface area (TCSA)	summation of surface areas of all calcifications of carotid plaques in each subject
ratio in total volume between calcifications and plaques (RTVCP)	the ratio between TCV and TPV in each subject
RTSACP	ratio in total surface area between calcifications and plaques
PVSARmax	maximum of the ratio between plaque volume and surface area among all plaques of each subject
PVSARmean	mean value of the ratio between plaque volume and surface area among all plaques of each subject
PVSARmin	minimum of the ratio between plaque volume and surface area among all plaques of each subject
PVSARm	the ratio between plaque volume and surface area of the largest plaque (i.e., with maximal volume) in each subject

Spearman rank correlation analysis was used to analyze the relationship between the plaque- and calcification-related parameters, and with hematological features. For quantitative data, the normal distribution was examined using Shapiro-Wilk or Kolmogorov–Smirnov tests when sample size ≤ 50 and >50 respectively [25]. When the normal distribution was satisfied (defined as *P* > 0.05 in Shapiro-Wilk or Kolmogorov–Smirnov test) and violated, the quantitative data were expressed by mean ± standard deviation (SD) and M (P25, P75) respectively, where M, P25, and P75 present the 50, 25, and 75 percentiles. The counting data were expressed as n (%). For binary features (i.e., gender, hypertension, and diabetes), the values of geometric parameters or their secondary parameters were compared between two subgroups using independent sample *t*-test (if normal distribution was satisfied in both subgroups) or Mann-Whitney *U* test (when normal distribution was violated in any subgroup).

Analysis of variance (ANOVA) or its non-parametric counterpart (Scheirer–Ray–Hare test) was performed on plaque volume with subject number and side (i.e., left vs. right carotid arteries) as independent categorical variables to investigate if plaque size was subject- or/and side-dependent. Levene's test was performed to investigate if the data satisfied the homogeneity of variance (defined as *P* > 0.05). ANOVA and Scheirer–Ray–Hare test were performed when the hypothesis of homogeneity of variance was satisfied and violated, respectively.

## 3 Results

### 3.1 General information

Table 2 lists the information of the 42 subjects. In total, 112 atherosclerotic plaques and 74 calcifications were extracted. There were 64 plaques within left carotid arteries, of which 52 were located at or around the bifurcation between external and internal carotid arteries (i.e., carotid sinus). There were 48 plaques within right carotid arteries, of which 20 were located at or around the bifurcation. Of all the subjects, the numbers of plaques and calcifications were 2.67 ± 1.87 and 1.76 ± 1.32.

**Table 2:** Baseline data characteristics of subjects.

Features	Values	Data Source
Gender Male [case (%)]	28 (66.7)	General information
Age	67.29 ± 6.33	General information
Hypertension [case (%)]	32 (76.2)	Medical history
Diabetes [case (%)]	7 (16.7)	Medical history
HDL-C [M (P25, P75), mmol/L]	1.14 (1.03, 1.33)	Blood test
LDL-C (mmol/L)	2.81 ± 0.91	Blood test
TC [M (P25, P75), mmol/L]	4.34 (3.84, 4.94)	Blood test
TG [M (P25, P75), mmol/L]	1.32 (0.86, 1.96)	Blood test
FOL [M (P25, P75), nmol/L]	13.37 (8.38, 25.55)	Blood test
VB12 [M (P25, P75), pmmol/L]	309.50 (141, 484)	Blood test
FIB (g/L)	3.87 ± 0.86	Blood test
UA (ummol/L)	322.52 ± 105.69	Blood test
TPV (mm^3)	443.45 (296.77, 741.72)	Geometric analysis
MPV (mm^3)	272.64 (147.25, 396.37)	Geometric analysis
TPSA (mm^2)	564.86 (360.83, 880.02)	Geometric analysis
MPSA (mm^2)	301.41 (212.06, 411.10)	Geometric analysis
TCV (mm^3)	49.23 (9.62, 190.91)	Geometric analysis
TCSA (mm^2)	128.05 (31.41, 222.95)	Geometric analysis
RTVCP	0.14 (0.02, 0.25)	Geometric analysis
RTSACP	0.25 (0.08, 0.33)	Geometric analysis
PVSARmax (mm)	0.83 (0.67, 1.01)	Geometric analysis
PVSARmean (mm)	0.75 (0.63, 0.87)	Geometric analysis
PVSARmin (mm)	0.66 (0.57, 0.73)	Geometric analysis
PVSARm (mm)	0.82 (0.6, 1.00)	Geometric analysis

Notes: TC: total cholesterol, TG: triglyceride, HDL-C: high-density lipoprotein cholesterol, LDL-C: low-density lipoprotein cholesterol, FIB: fibrinogen, UA: uric acid, TPV: total plaque volume, MPV: maximum plaque volume, TPSA: total plaque surface area, MPSA: maximal plaque surface area, TCV: total calcification volume, TCSA: total calcification surface area, RTVCP: ratio in total volume between calcifications and plaques, RTSACP: ratio in total surface area between calcifications and plaques, PVSARmax: maximum of the ratio between plaque volume and surface area, PVSARmean: mean value of the ratio between plaque volume and surface area, PVSARmin: minimum of the ratio between plaque volume and surface area, PVSARm: the ratio between plaque volume and surface area of the largest plaque (i.e., the plaque with maximal volume).

### 3.2 Plaque-based analysis

Significant linear correlations were globally observed between multidimensional geometric parameters of carotid plaques (R-squared >0.25 and *P* < 0.05 for all pairs), in which strong linear correlations existed among the following pairs: volume and surface area (R-squared = 0.88), volume and cross-sectional area (R-squared = 0.80), surface area and length (R-squared = 0.67), surface area and surface distance (R-squared = 0.77), surface area and cross-sectional area (R-squared = 0.72), as well as length and surface distance (R-squared = 0.82).

**Fig. 2:**
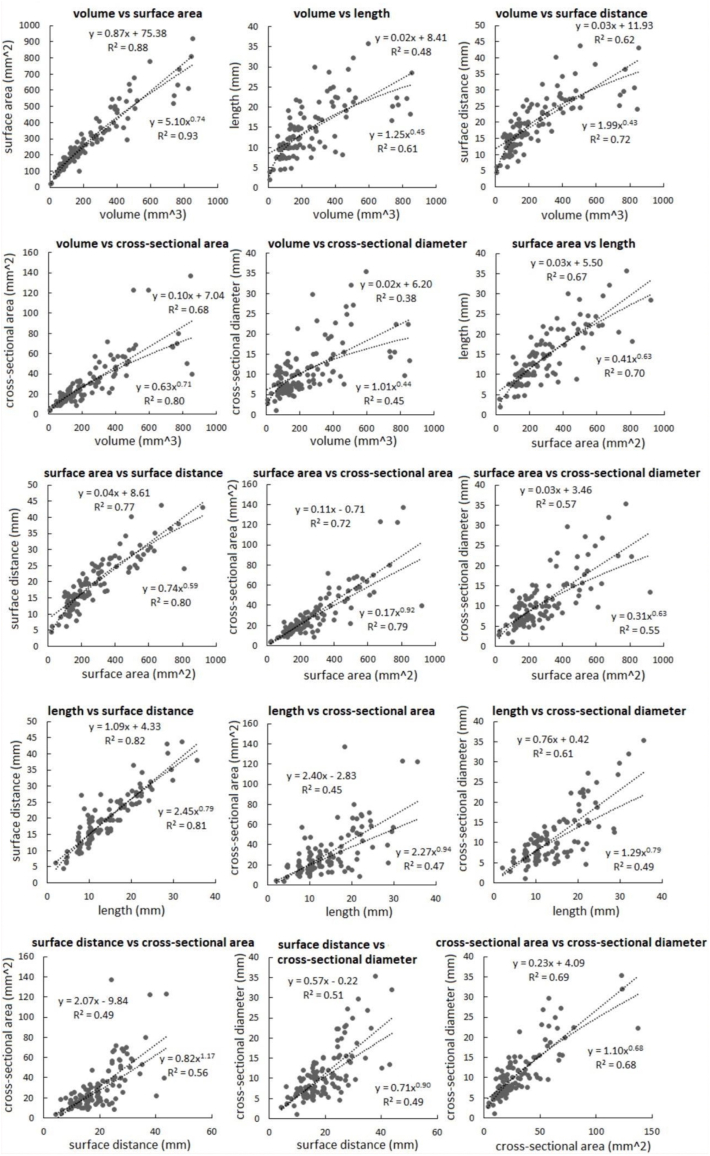
Linear and non-linear regression fittings on multidimensional geometric parameters of carotid plaques.

The non-linear regression showed a higher R-squared value than the corresponding linear regression in the following pairs (Fig. 2): volume and surface area (R-squared values in non-linear and linear regressions: 0.93 vs 0.88), volume and length (0.61 vs 0.48), volume and surface distance (0.72 vs 0.62), volume and cross-sectional area (0.80 vs 0.68), volume and cross-section diameter (0.45 vs 0.38), surface area and length (0.70 vs 0.67), surface area and surface distance (0.80 vs 0.77), surface area and cross-sectional area (0.79 vs 0.72), length and cross-sectional area (0.47 vs 0.45), as well as surface distance and cross-sectional area (0.56 vs 0.49).

The ratio between the upstream and downstream lengths was correlated significantly with the length (Spearman's rank correlation coefficient: 0.197, *P* = 0.037) but not the volume (Spearman's rank correlation coefficient: 0.155, *P* = 0.103).

**Fig. 3:**
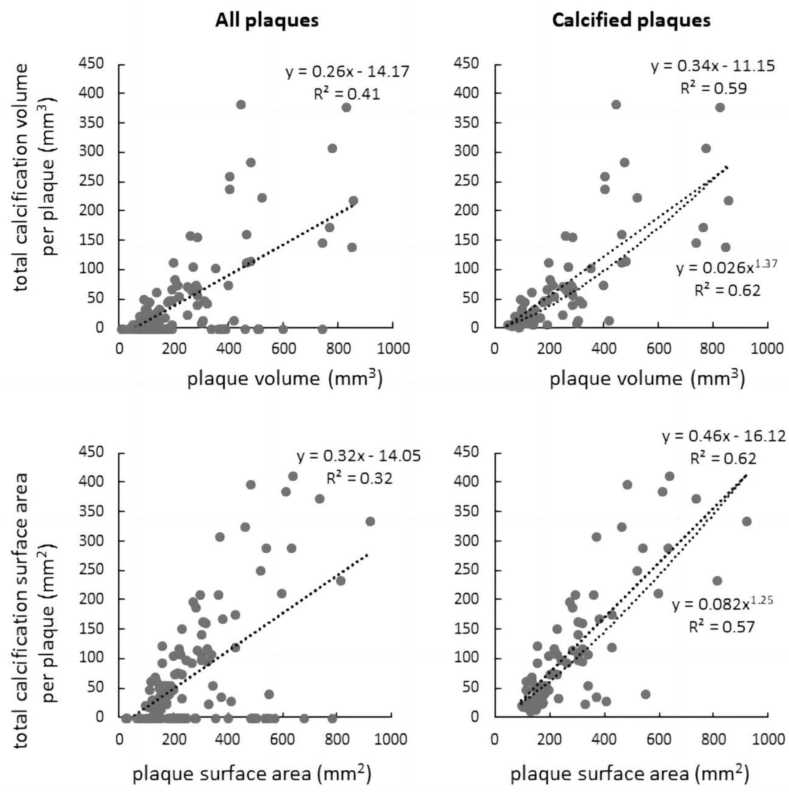
Linear and non-linear regressions between plaques and total calcifications per plaque in volume and surface area.

Among all plaques, the plaque volume was correlated significantly with the total volume of intra-plaque calcifications (Spearman's rank correlation coefficient: 0.468, *P* < 0.001), similarly as between plaque surface area and the total surface areas of intra-plaque calcifications (Spearman's rank correlation coefficient: 0.402, *P* < 0.001). The relationships were even more significant when non-calcified plaques were excluded (for volume: 0.785, *P* < 0.001; for surface area: 0.741, *P* < 0.001). Compared with linear fitting, the non-linear trend was observed in volume but not surface area (Fig. 3).

### 3.3 Subject-based analysis

In subject-based analysis, the TPV was significantly correlated with the following plaque-related parameters: MPV (Spearman's rank correlation coefficient: 0.740, *P* < 0.001), TPSA (0.952, *P* < 0.001), MPSA (0.729, *P* < 0.001), following calcification-related parameters: TCV (0.692, *P* < 0.001), TCSA (0.711, *P* < 0.001), as well as following secondary parameters: RTVCP (0.400, *P* = 0.009), PVSARmax (0.531, *P* < 0.001), PVSARmean (0.377, *P* = 0.014), PVSARm (0.387, *P* = 0.011).

Regarding the hematological features, TC was significantly correlated with the following geometric and secondary parameters: TPV (0.356, *P* = 0.021), MPV (0.373, *P* = 0.015), TPSA (0.362, *P* = 0.018), MPSA (0.388, *P* = 0.011), and marginally with TCV (0.299, *P* = 0.054) and TCSA (0.304, *P* = 0.051). TG was significantly correlated with TPV (0.499, *P* = 0.001), MPV (0.346, *P* = 0.025), TPSA (0.536, *P* < 0.001), MPSA (0.315, *P* = 0.042), TCV (0.400, *P* = 0.009), TCSA (0.382, *P* = 0.013). FOL was significantly negatively correlated with TPV (−0.327, *P* = 0.034) and TPSA (−0.375, *P* = 0.014). VB12 was significantly correlated with TPV (0.298, *P* = 0.055). LDL-C was significantly correlated with MPSA (0.313, *P* = 0.044).

The results of *t*-test and Mann-Whitney *U* test indicated no significant difference in geometric and secondary parameters between male and female subjects, or between subjects with and without hypertension or diabetes (*P* > 0.05 for all).

The homogeneity of variance was violated in the distribution of plaque volume on left and right sides (*P* < 0.05 in Levene's tests). Therefore, the Scheirer–Ray–Hare test was performed. There was a marginally significant influence of subject on plaque size (*P* = 0.046), without any significant influence of the side, or the interaction between side and subject (*P* > 0.05 for both).

## 4 Discussion

### 4.1 Summary of findings

We observed a consistency in geometry among carotid atherosclerotic plaques of different sizes, while the plaque size could be significantly different among subjects. Significant correlations widely existed among multidimensional geometric parameters with non-linear relationships. The lipid profile was associated with the size of carotid plaque.

### 4.2 Evolution of carotid plaque geometry

We observed that the ratio between upstream and downstream segments of a plaque was associated with plaque size, indicating a certain pattern of elongation during plaque growth. The geometric-hemodynamic-biochemical interaction shapes carotid plaque growth [26,27], leading to a higher frequency of eccentric and longitudinally asymmetrical (often distally-extended) plaques [28].

The carotid plaques can form at young ages and undergo complex evolution during aging, where many factors including lifestyle, accompanying diseases, and genetics are involved [29]. Whereas, the evaluation of carotid atherosclerosis is often neglected until 50s–60s when cardiovascular disorders and symptoms appear. Current research on carotid plaque geometry is focused on the clinical indications, e.g., the risk of stroke [5,6,13,30]. The evolution of carotid plaque geometry is underinvestigated, partly due to the scarcity of imaging data on asymptomatic carotid plaques. We selected the cases in mild to moderate stenosis without lipid control or other clinical intervention, which enabled us to observe carotid plaque geometry under natural evolution. As far as we know, this is the first quantitative analysis on carotid plaque geometry using multidimensional parameters.

### 4.3 Calcification and dyslipidemia in early-stage carotid atherosclerosis

The subject-based analysis revealed strong geometric relationships between plaque and calcification (Fig. 3). Calcification appears at later stages of atherosclerosis. The early- and late-stage plaques differ in components and morphology. We observed that the non-linearity of the relationships between geometric parameters are more significant in large plaques (Fig. 2), which indicated the change of growth pattern along the development of carotid plaques. The subjects in this study are mainly old people (minimal age: 52 years). Therefore, the carotid plaques, even the small ones, might had been developing for years, which enables the development of calcification. The calcification in early-stage carotid plaques warrants further investigation, where the scarcity of radiological data in young subjects is a major challenge.

Among the hematological features, TG was significantly correlated with most plaque geometric parameters, followed by TC and LDL-C. These results highlight the need for long-term monitoring of dyslipidemia in investigating the evolution of carotid plaque geometry.

### 4.4 Differences between carotid and coronary plaques: biomechanical and hemodynamic factors

It has been observed that non-linear relationships exist among multidimensional geometric parameters of coronary plaques [23]. We found these relationships are stronger in carotid plaques, as demonstrated by the R-squared values in Fig. 2 in contrast with Fig. 3 of [23]. These results indicated higher geometric consistency in carotid plaque than coronary plaques.

As large elastic arteries, carotid and coronary arteries share similar vasomotor functions and anatomical structures, while their biomechanical and hemodynamic environments are totally different. Firstly, carotid blood flow is antigravity during almost two-thirds of the day when the individual is standing or sitting, while coronary flow is to be with the gravity [9]. Secondly, coronary arteries, especially left anterior descending (LAD) artery, undergo cyclic bending motion and myocardial contraction, leading to an unusual flow curve where inflow maximizes during diastole. As a result, mean WSS in normal human carotid artery (1.1–1.3 Pa) could be higher than that of normal coronary artery (0.68 Pa, range 0.33–1.24 Pa) [31]. Considering the atherogenetic role of low WSS, the higher WSS value of carotid arteries might partly explain the observation that carotid atherosclerosis develops later in life compared to coronary artery disease [32]. Due to the complex biomechanical and hemodynamic environment, coronary plaque formation involves more factors including the dynamics of local coronary artery as well as the length and depth of the bridged segment (i.e., the artery segment embedded in cardiac muscles, which can significantly decrease the WSS in its proximal segment) [33,34]. In comparison, the carotid flow is stabilized by cerebral autoregulation, which may induce a more regular pattern of plaque growth.

Some mathematical models have been proposed to simulate the growth of carotid plaques based on idealized geometry model or small cohorts [[35], [36], [37]]. The computational complexity and the workload of plaque segmentation limit the sample size of patient-specific models. The observed geometric consistency among different plaques highlighted the need for large-scale investigation. The results also provided quantitative reference for geometric modelling of carotid plaques in biomechanical simulation.

### 4.5 Carotid plaque reconstruction: towards higher accuracy and automation

Some new algorithms have been proposed for quantitative evaluation of carotid plaque geometry (e.g., total volume, stenosis in diameter, and stenosis in area) and tissue composition using CTA images [38]. Compared with MRI, the advantages of CT include high spatial resolution, fast acquisition, broad availability, as well as direct visualization of both luminal stenosis and calcifications [39]. However, the CTA images acquired in clinical settings often have low spatial resolution and are affected by blooming artefact [40]. Accurate quantification of carotid plaques with irregular surface geometry is also challenging in MRI [41]. Therefore, semiautomatic plaque extraction with essential manual modification is widely used [23,30,38]. The non-linear relationships between multidimensional geometric parameters can provide prior knowledge for carotid plaque segmentation, which may improve the reliability and efficiency of automatic quantification of irregular plaques [42,43].

### 4.6 Clinical indication of carotid plaque geometry

The geometric features of carotid plaques including surface irregularities [13] and the length of upstream segment [11] are associated with plaque vulnerability and the risk of stroke, which might originate from the biochemical and apoptotic changes induced by local hemodynamic environment [15]. The total plaque area and plaque thickness can enhance risk stratification [44]. The results of ultrasound measurement showed that carotid plaque geometry had a higher diagnostic accuracy for the prediction of future CAD events, compared to carotid intima-media thickness [10]. A linear relationship between mechanically unstable areas of carotid plaques and cognitive decline suggests a possibly contributory role of microemboli from mechanically unstable carotid plaques for “silent strokes” [45]. Currently, the machine learning methods for automatic plaque classification rely on cross-validation of ground truth, i.e., supervised learning [46]. The consistency of plaque geometry may provide a new approach of automatic plaque classification and risk stratification by evaluating the deviation of a plaque from the normal geometric patterns.

### 4.7 Limitations and future directions

This study has some limitations. First, only 42 patients were finally included. The result of Scheirer–Ray–Hare test indicated that plaque size might be significantly different between subjects, while the intra-subject differences between plaques warrant further investigation. Carotid CTA scan is uncommon in asymptomatic patients, which largely limits the sample size. Secondly, there was no follow-up imaging data. The longitudinal observation of plaque geometry can reveal the trend of plaque growth and validate our findings. Finally, current study is focused on plaque geometry, without including the anatomy and geometry of carotid arteries, as well as traditional risk factors [47].

In the future, multicenter studies are essential for large-scale validation and in-depth investigation on carotid plaque geometry in different cohorts and on intra-subject differences in plaque geometry. Long-term follow-up can be considered to directly observe the evolution of different carotid plaques and validate our findings. Regarding the geometric consistency, the relationships between multidimensional parameters can be compared between symptomatic and asymptomatic plaques [48]. With high-quality imaging data, the geometric consistency in calcifications and fibrous tissues can be investigated based on existing observation [49]. Finally, geometric features of local carotid artery and other risk factors can be investigated to disclose the interaction between carotid plaques and its biomechanical environment.

## 5 Conclusion

There is a consistency in geometry among carotid atherosclerotic plaques of different sizes. The size of a carotid plaque is associated with the patient's lipid profile.

## Funding

This research was funded by Medical and Health Science and Technology Plan Project of Zhoushan and the Science and Technology Project of Zhoushan, China, grant number 2021YA07, 2020C31048.

## Data availability statement

The data that support the findings of this study are available on request from the corresponding author.

## CRediT authorship contribution statement

**Ling Li:** Conceptualization, Data curation, Funding acquisition, Investigation, Methodology, Software, Writing – original draft. **Fangyu Dai:** Conceptualization, Methodology, Project administration, Supervision, Writing – review & editing. **Jie Xu:** Data curation, Investigation, Methodology, Software, Validation, Writing – review & editing. **Jiaoxuan Dong:** Data curation, Investigation, Methodology, Visualization, Writing – review & editing. **Bin Wu:** Investigation, Resources, Software, Visualization, Writing – review & editing. **Songbin He:** Funding acquisition, Supervision, Writing – review & editing. **Haipeng Liu:** Conceptualization, Investigation, Methodology, Project administration, Resources, Supervision, Writing – review & editing.

## Declaration of competing interest

The authors declare that they have no known competing financial interests or personal relationships that could have appeared to influence the work reported in this paper.
